# Artificial intelligence enabled smart phone app for real-time caries detection on bitewing radiographs

**DOI:** 10.6026/973206300200243

**Published:** 2024-03-31

**Authors:** Nupur Dhanak, Vaibhav T Chougule, Keerthi Nalluri, Ankur Kakkad, Ankit Dhimole, Anuj Singh Parihar

**Affiliations:** 1 Department of Conservative Dentistry and Endodontics, Government Dental College and Hospital, Ahmadabad, Gujarat, India; 2 Department of Paediatric and Preventive Dentistry, Bharati Vidyapeeth (Deemed to be University) Dental College and Hospital, Sangli, Maharashtra, India; 3 Apex, North Carolina, USA; 4 Department of Oral Medicine and Radiology, Hitkarini Dental College and Hospital, Jabalpur, MP, India; 5 Department of Oral Medicine and Radiology, Hitkarini Dental College and Hospital, Jabalpur, MP, India; 6 Department of Periodontology, People's Dental Academy, Bhopal, Madhya Pradesh, India

**Keywords:** Artificial intelligence, bitewing radiograph, caries detection, mobile phone

## Abstract

Diagnosis of proximal caries is a difficult task. Artificial intelligence (AI) enabled diagnosis is gaining momentum. Therefore, it is of interest to evaluate the effectiveness of an artificial intelligence (AI) smart phone application for bitewing radiography towards real-time caries lesion detection. The Efficient Det-Lite1 artificial neural network was used after training 100 radiographic images obtained from the department of Oral Medicine. Trained model was then installed in a Google Pixel 6 (GP6) smartphone as artificial intelligence app. The back-facing mobile phone video camera of GP6 was utilised to detect caries lesions on 100 bitewing radiographs (BWR) with 80 carious lesion in real-time. Two different techniques such as scanning the static BWR on laptop with a moving mobile and scanning the moving radiograph on the laptop with stationery mobile were used. The average value of sensitivity/precision/F1 scores for both the techniques was 0.75/0.846 and 0.795 respectively. AI programme using the rear-facing mobile phone video camera was found to detect 75% of caries lesions in real time on 100 BWR with a precision of 84.6%. Thus, the use of AI with smart phone app is useful for caries diagnosis which is readily accessible, easy to use and fast.

## Background:

One of the most prevalent chronic illnesses in the world is dental caries [1]. Despite being advised as a diagnostic technique, radiography might be subjective in its ability to identify dental caries [2]. The gold standard for identifying proximal caries lesions is currently bitewing radiography (BWR). Interpreting BWR, however, remains subjective for a variety of reasons. Magnification, sharpness, distortion, and target/object receptor distance and alignment can all be impacted by focal size, movement, film composition, and density and contrast [3]. Artificial intelligence (AI) technologies and computer-aided image analysis tools have been developed to help in this regard [2, 4]. The ability of computer-based diagnosis to identify lesions and caries that are invisible to the human eye is why it is becoming more and more popular. Deep learning (DL), adaptive neural network design, artificial multilayer perceptron neural network, convolutional neural network (CNN), back-propagation neural network, and k means clustering are some of the methods used for caries detection [5]. With a quick download, a dentist might turn a smartphone into a caries detector, potentially enabling worldwide deployment of an app-based caries detector [2]. AI-enabled software and services are currently available from companies like Pearl AI, Dentrix (VideaHealth), and Carestream to assist dentists in diagnosing radiographic caries. However, limitation in adoption of these technologies makes the difficulties in implementing it for clinical usage. The suggested dental diagnostic tool would have to be quick, simple, and easy to use [2]. Therefore, it is of interest to describe the use of an artificial intelligence enabled smart phone app for real-time caries detection on bitewing radiographs.

## Materials and Method:

## Study design:

We retrieved 100 bitewing radiographs (BWR) with carious lesions from Department of Oral Medicine and Radiology, Hitkarini Dental College and Hospital, Jabalpur, India. A total of 80 carious lesions found in 100 bitewing radiographs were included for training and validation of an EfficientDet-Lite1(EDL1, Google, Mountain View, CA, USA) artificial intelligent powered object detection software. Incipient carious lesions were not included in the training. Trained model was deployed on Google Pixel 6(GP 6) phone to detect the caries which were displayed on lap top. Performance of AI based app was evaluated by testing 100 new BWR with 80 carious lesions. Images of bitewing radiographs were converted to JPG format. All photos were shrunk to 384 x 384 pixels using the AI model before any inference was made. Only pictures with clearly visible, clinically curable caries lesion were included in the study. These pictures had an average size of 500 KB. Artifacts were not annotated to avoid their effect on training of the software. Caries detection was done based on Dr. Joen Iannucci's Radiographic Caries Identification (RCI) method [7].

Five classes were created to categorise carious lesions [2]:

[1] Enamel: proximal caries that are only apparent in the enamel

[2] Dentin: obvious interproximal cavities within the dentin.

[3] Pulp: Caries that are close to or reach the pulp chamber

[4] Recurrent: Caries under existing restorations

[5] Occlusal: Caries under the occlusal surface, including superimposed buccal or lingual /palatal caries

## Methodology:

AI and smart phone caries detection was accomplished using the Jim Pun *et al.* methods. The investigator modified all of the open-source software used in this study to get the desired functionality. The investigator developed a unique caries detector using the Tensor Flow ML framework (TF, Google, Mountain View, CA, USA) [2]. Because TF Lite (TFL, Google, Mountain View, CA, USA) featured more compact and effective TF models, the investigator chose to use it for mobile phone distribution. The investigator selected the lightweight EDL1 ANN model, which is based on the Efficient Det neural network architecture, in order to maximise performance on the GP6 mobile. EDL1 ANN is an open source network that requires no core programming modifications.

The TFL Model Maker libraries (Google, Mountain View, CA, USA) were run in a virtual Ubuntu OS environment on a Lenova desktop computer and Jupyter notebook for training and validation. Using an eight-batch size, the model was trained for 30, 40, and 70 epochs. The best trained model was determined by taking the highest average precision at 0.50 Intersection over Union (IoU, AP50), which corresponds to 40 epochs.

On the mobile phone, post-training quantization was utilised to lower latency and memory footprint. To minimise the size of the model, floating-point representations are quantized by converting them to fixed integer values. Later Android Studio Electric Eel (ASEE, Google, Mountain View, CA, USA) received the quantized model. The trained model was integrated in an object detection starting programme that ASEE created and installed on the GP6 for testing.

Mac Book Pro with 14" display was used to display BTW radiographic images. Portrait mode of GP6 was used for scanning the BWR which permitted only a portion of a BWR to be scanned at once. In order to scan the full BWR, either the phone or the BWR has to be moved from end to end. Therefore, two testing techniques were adapted: scanning the static BWR on laptop with a moving mobile and scanning the moving radiograph on the laptop with stationery mobile. In first technique, GP6 took average 20secs to scan the image on laptop. For the second technique, IMovie was used to move radiograph from left to right in 40secs, while GP6 was held stationery to record this. In both the techniques, video was captured using in built video recorder of GP6 mobile phone.

With the detection threshold set to 0.4, the GP6 was able to identify caries using its back-facing video camera. Bounding boxes would appear and go in real time as the detections also changed, increasing the cumulative detection as the images in the video or the handheld static radiograph detection altered. The real-time detections were captured by the GP6's native video recorder for examination at a later time. After being moved to the MacBook, iMovie was used to import the film and count frames by frame. The identical caries lesion found in many frames was considered a duplicate detection, which was disregarded. Likewise, the same lesion that was identified as "pulp" in one frame and "dentin" in another as well as similar detections of various classes were disregarded.

## Metrics:

The trained model included the following detection definitions for GP6 real-time video testing:

True positive (TP): the quantity of accurate diagnoses made when there is actual caries.

False positive (FP): the quantity of false positives when there are no actual cavities.

False negative (FN): the quantity of missed detections when actual caries is present.

The performance metrics listed below were also provided:

Sensitivity (Recall, True Positive Rate (TPR)) = TP/(TP + FN).

Precision (Positive Predictive Value (PPV)) = TP/(TP + FP).

F1 Score = 2TP/(2TP + FP + FN).

## Results:

Results of the study are tabulated in Table 1. In the first method, the portable GP6 moved over static BWR on laptop to identify caries. 64 out of 80 caries lesions were recognised cumulatively by the GP6 after tabulating each video frame. Rest 16 lesions were not detected and thus were false negative. 7 false positive lesions were noted by this technique. Sensitivity/Precision/F1 score for first method was 0.775/0.838 and 0.805 respectively. In the second method, GP6 was stationery and recording was taken of moving BWR on laptop screen. It detected, 58 caries lesions. 22 lesions went undetected and 10 false positive lesions were noted. Sensitivity/Precision/F1 scores for this group were 0.725/0.853 and 0.784 respectively. Mean Sensitivity/Precision/ F1 Score of both the techniques adapted was 0.75/0.846 and 0.795 respectively. Thus, the AI programme using the back-facing mobile phone video camera could detect 75% of caries lesions on 50 BWR in real time with a precision of 84.6%, based on an average of the aggregate results.

## Discussion:

Artificial Neural Network (ANN) involves computations and mathematics, which simulates human brain processes. A modelling research has determined that artificial intelligence is a cost-effective method for caries diagnosis on radiographs [14]. However, such software requires substantial memory and computing power to conduct complex task. These days advances in mobile technology have made it possible to deploy such smart app in mobile. We utilised an AI programme that ran on a mobile device. ANN requires training using a large dataset of labelled images, where the network learns to recognize features associated with specific objects, in our case dental caries on bitewing radiographs. In present study 100 BWR with 220 carious lesions were augmented to 1584(90%) and 176 (10%) lesions respectively for training and validation purpose. Once open, this software works similarly to the popular face-tracking feature on smartphones - it just tracks dental cavities instead of faces. It is not necessary for the user to take separate pictures of the BWR for detection because detections were carried out in real-time video. This eliminates the necessity of taking photos and continuously tapping the screen to record specific frames for later inference. The third speed feature was met by the experimental app, which ran on the GP6, which generated inferences in real time at an estimated 14 FPS, or roughly 71 mS per inference frame. Newer mobile phones will accelerate inference, leading to even faster detections [2].

In present study, we used radiographs of patients and not from internet. This reduces artifacts such as low resolution, lines, annotations, etc. A comparison of earlier research on AI-based caries detection is shown in Table 2. We obtained improved sensitivity and precious scores in comparison to earlier investigations by Jim Un *et al.* [2] and Lee *et al.* [1]. We found that the first method detected true positive at the cost of slightly more false positive, thus greater sensitivity (77.5%) and lower precision (83.8%) compared to second method having precision of 85%. This could be because of greater variations in movement of mobile in 1st technique. This finding is in agreement with study by Jim Pun *et al.*[2] For both the methods, cervical burnout was the main reason for false-positive detection.

In sensitivity/precision/F1 scores comparison between the real-time video results and static-detection tests on non-mobile phones, Srivastava *et al.* obtained 0.805/0.615/0.7scores [8]. "Very accurate performance" was reported by Lee *et al.* with scores of 0.65/0.633/0.641 [1]. Kunt *et al.* put together a sizable annotated bitewing radiograph dataset and employed convolutional neural networks to automatically detect dental caries in bitewing radiographs with performance comparable to that of a person. They came to the conclusion that the trained ensemble of object detection CNNs performed at least as well as seasoned dentists in detecting caries with a fair level of accuracy. Inconsistencies in the training dataset probably hampered the performance on tiny lesions [15]. According to Schropp *et al.* dental students' ability to identify proximal enamel caries in bitewing radiographs was unaffected by training with the AI programme [4].

Tichý *et al.* examined the automatic method's ability to detect caries in bitewing radiographs from multiple dentists and assessed its efficacy in detecting caries in the absence of a trustworthy ground. They came to the conclusion that the automatic approach consistently beat dentists with extensive expertise and excelled novices [16]. Zheng *et al.* came to the conclusion that the CNN of ResNet18 performed well in diagnosing pulpitis and deep caries [11]. Geetha *et al.* used adaptive threshold segmentation on a back-propagation neural network model using 105 images to achieve nearly flawless scores of 0.962/0.963/0.962 [9]. Cantu *et al.* came to the conclusion in their study that a deep neural network was substantially more accurate than dentists in detecting caries lesions on bitewing radiographs [6]. Dental caries in bitewing radiographs may be precisely and successfully detected and segmented by CNN-based AI algorithms [12]. Thus results of various studies comply with our study, proving ability of artificial intelligence to detect proximal carious lesions with acceptable precision. The very modest number of useable radiographs with caries is a limitation of the current investigation. Also, further studies with better performing CNN model could be done for better precision.

## Conclusion:

Data shows that AI with a smartphone app to diagnose dental caries is comparatively simple, quick, and promising. Using the rear-facing smartphone video camera, an AI programme identified 75% of caries lesions on 50 BWR in real time with a precision of 84.6%.

## Figures and Tables

**Table 1:** Mean performance of AI with smart phone app using real-time caries detection

	**Total caries**	**True positive TP**	**False positive FP**	**False negative FN**	**Sensitivity TP/(TP+FN)**	**Precision TP/(TP+FP)**	**F 1 score 2TP/(2TP + FP + FN)**
Stationery Image, Moving Mobile*	80	62	12	18	0.775	0.838	0.805
Moving Image, Stationery Mobile*	80	58	10	22	0.725	0.853	0.784
Mean					0.75	0.846	0.795
* Aggregate detection

**Table 2:** Comparison of published studies on AI in caries detection

**Studies on AI in caries detection**	**Sensitivity TP/(TP + FN)**	**Precision TP/(TP + FP)**	**F1 Score 2TP/(2TP + FP + FN)**
Srivastava*et al.* 2017 [8]	0.805	0.615	0.7
Geetha*et al.* 2020 [9]	0.962	0.963	0.962
Lee*et al.* 2021 [1]	0.65	0.633	0.641
Vinayahalingam*et al.* 2021 [10]	0.86	0.88	0.86
Zheng *et al* 2021 [11]	0.89	0.85	0.86
Bayraktar*et al.* 2022 [12]	0.84	0.84	0.84
Jim Pun*et al.* 2023 [2]	0.625	0.706	0.661
Albano D*et al.*2024 [13]	0.86	0.94	0.92
Dhanak*et al.* (Our study result) 2024	0.75	0.846	0.795
